# Fetoscopic endoluminal tracheal occlusion and reestablishment of fetal airways for congenital diaphragmatic hernia

**DOI:** 10.1186/s10397-018-1041-9

**Published:** 2018-05-08

**Authors:** Lennart Van der Veeken, Francesca Maria Russo, Luc De Catte, Eduard Gratacos, Alexandra Benachi, Yves Ville, Kypros Nicolaides, Christoph Berg, Glenn Gardener, Nicola Persico, Pietro Bagolan, Greg Ryan, Michael A. Belfort, Jan Deprest

**Affiliations:** 1 Academic Department of Development and Regeneration, Woman and Child, Biomedical Sciences, and Clinical Department of Obstetrics and Gynaecology, KU Leuven, Herestraat 49, 3000, Leuven, Belgium; 2 TOTAL (Tracheal Occlusion To Accelerate Lung Growth Trial) Consortium, Leuven, Belgium; 3 BCNatal – Barcelona Center for MaternaleFetal and Neonatal Medicine (Hospital Clínic and Hospital Sant Joan de Déu), IDIBAPS, University of Barcelona, and Centre for Biomedical Research on Rare Diseases (CIBER-ER), Barcelona, Spain; 4 Department of Obstetrics, Gynaecology and Reproductive Medicine, Hôpital Antoine-Béclère, University Paris Sud, Clamart, France; 5 Fetal Medicine Unit, Obstetrics and Fetal Medicine Department, Necker-Enfants Malades Hospital, Université Paris Descartes, Sorbonne Paris Cité, Paris, France; 6 Harris Birthright Centre, King’s College Hospital, London, UK; 7 Division of Fetal Surgery, Department of Obstetrics and Prenatal Medicine, University of Bonn, Bonn, Germany; 8 Department of Obstetrics and Gynecology, University of Cologne, Cologne, Germany; 9 Mater Health Services, Mater Research UQ, Brisbane, Australia; 10 Department of Obstetrics and Gynecology, “L. Mangiagalli,” Fondazione IRCCS “Ca’ Granda” - Ospedale Maggiore Policlinico, Milan, Italy; 11 Neonatal Surgery Unit, Department of Medical and Surgical Neonatology, Bambino Gesù Children’s Hospital, IRCCS, Piazza S. Onofrio, 4, 00165 Rome, Italy; 12 Fetal Medicine Unit, Mt Sinai Hospital, University of Toronto, Toronto, Canada; 13 Department of Obstetrics and Gynecology, Baylor College of Medicine and Texas Children’s Fetal Center, Houston, Texas USA; 14 European Reference Network on Rare and Inherited Congenital Anomalies “ERNICA”, Rotterdam, The Netherlands

**Keywords:** FETO, Fetal endoluminal tracheal occlusion, CDH, Congenital diaphragmatic hernia, Fetal surgery, Fetoscopy

## Abstract

**Background:**

Congenital diaphragmatic hernia (CDH) is a congenital anomaly with high mortality and morbidity mainly due to pulmonary hypoplasia and hypertension. Temporary fetal tracheal occlusion to promote prenatal lung growth may improve survival. Entrapment of lung fluid stretches the airways, leading to lung growth.

**Methods:**

Fetal endoluminal tracheal occlusion (FETO) is performed by percutaneous sono-endoscopic insertion of a balloon developed for interventional radiology. Reversal of the occlusion to induce lung maturation can be performed by fetoscopy, transabdominal puncture, tracheoscopy, or by postnatal removal if all else fails.

**Results:**

FETO and balloon removal have been shown safe in experienced hands. This paper deals with the technical aspects of balloon insertion and removal. While FETO is invasive, it has minimal maternal risks yet can cause preterm birth potentially offsetting its beneficial effects.

**Conclusion:**

For left-sided severe and moderate CDH, the procedure is considered investigational and is currently being evaluated in a global randomized clinical trial (https://www.totaltrial.eu/). The procedure can be clinically offered to fetuses with severe right-sided CDH.

**Electronic supplementary material:**

The online version of this article (10.1186/s10397-018-1041-9) contains supplementary material, which is available to authorized users.

## Background

Congenital diaphragmatic hernia (CDH) is a life-threatening condition affecting up to 3 in 10,000 live born babies [1]. The diaphragmatic defect allows abdominal organs to herniate into the thorax which prevents normal lung development. Depending on the side and size of the defect, this may be the liver, bowel, spleen, and/or stomach. The majority of defects are left sided (LCDH 85%). Thirteen percent are right sided (RCDH), and bilateral defects or other forms occur very rarely. Associated anomalies are frequent and should be ruled out by imaging and genetic testing as they independently influence survival and morbidity. In most registries currently, survival is approximately 70% depending on the case mix and location of treatment [2]. Surviving patients may suffer from not only medium and long-term morbidity predominantly respiratory in nature, but also gastro-esophageal reflux, failure to thrive, and less common orthopedic or other problems.

**Fig. 1:**
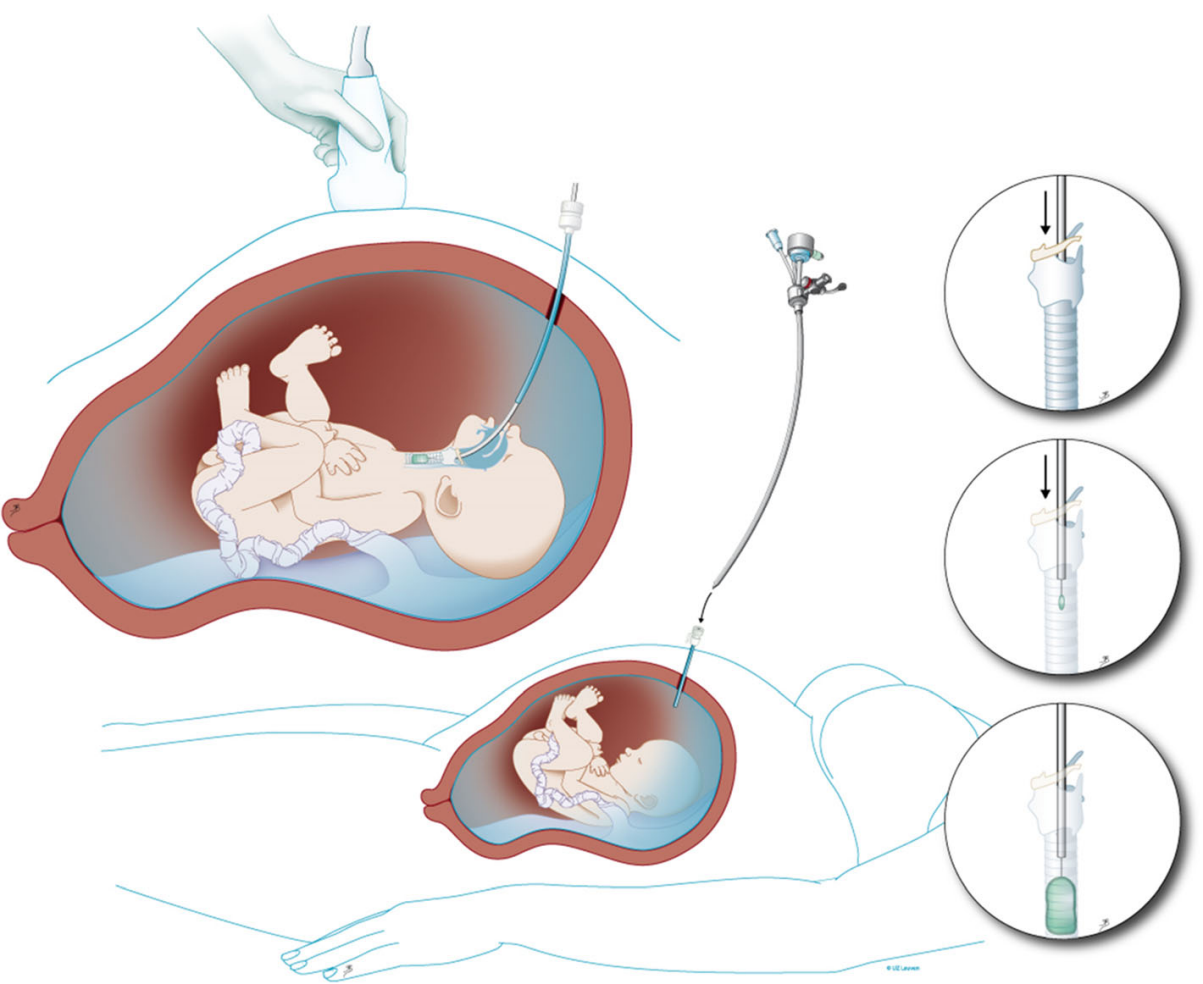
Fetal endoscopic tracheal occlusion (FETO): a schematic drawing showing access to uterus and the fetal trachea. Inserts: steps in balloon delivery. *© UZ Leuven, UZ Leuven, Belgium, drawing Myrthe Boymans*

Individualized prognosis of isolated CDH can be made prenatally by using the lung size, the presence of the liver in the thorax, and the side of the defect [3]. Patients with predicted poor prognosis can be offered experimental fetal therapy. To improve survival, the intervention should reverse pulmonary hypoplasia (i.e., stimulate lung growth) before birth. Historically, this was attempted by anatomical repair of the defect in utero. The results of this approach were suboptimal [4]. Also, the anatomic defect itself can be relatively easily managed after birth hence it is not the problem. An alternative strategy, based on the clinical observation that fetuses with laryngeal atresia have larger lungs, led to animal experiments that demonstrated that fetal tracheal occlusion reverses experimental pulmonary hypoplasia [5]. Tracheal occlusion leads to accumulation of lung fluid which in turn causes lung stretch. This activates a pathway that leads to proliferation and increased growth of the airways and pulmonary vessels, nicely summarized in the acronym PLUG: “Plug the Lung Until it Grows” (PLUG) [6]. However, when the occlusion is maintained until birth, the number of type II pneumocytes is abnormally low, leading to a relative surfactant deficiency [7]. By reversing the tracheal occlusion before birth, the balance between type I and type II pneumocytes at birth is more optimal. This is captured in the concept “plug-unplug sequence,” and reversal of occlusion is an important component in the fetal treatment strategy [7]. Nowadays, tracheal occlusion is uniquely performed by minimally invasive fetal endoluminal tracheal occlusion (FETO) and performed under sono-endoscopic guidance. This is a percutaneous procedure in which a latex balloon is endoscopically positioned above the carina and inflated to occlude the trachea [8] (Fig. 1). The present paper describes the instrumentation and the technical aspects of the FETO procedure as currently performed by the above clinical research consortium within their clinical trial.

## Methods

### Selection of fetuses

CDH is typically diagnosed during mid-gestation on screening ultrasound. Following the diagnosis (or suspicion thereof), patients are best referred to a tertiary center where this condition is routinely managed after birth. Patients will be reassessed with targeted ultrasound and magnetic resonance imaging (MRI) and will be offered genetic testing (today this is by micro-array analysis) [9]. This has two purposes, (1) to rule out associated structural or genetic anomalies, which themselves may worsen the prognosis dramatically and (2) to assess the severity of the pulmonary hypoplasia. This information is used to make a personalized prognosis and lead multidisciplinary counseling. Additional evaluation frequently reveals some elements of discordance with the initial assessment at the referring site and may well result in a changed perspective and different parental decisions [10]. Therefore, it is considered prudent to limit prognostic advice at first diagnosis.

**Fig. 2:**
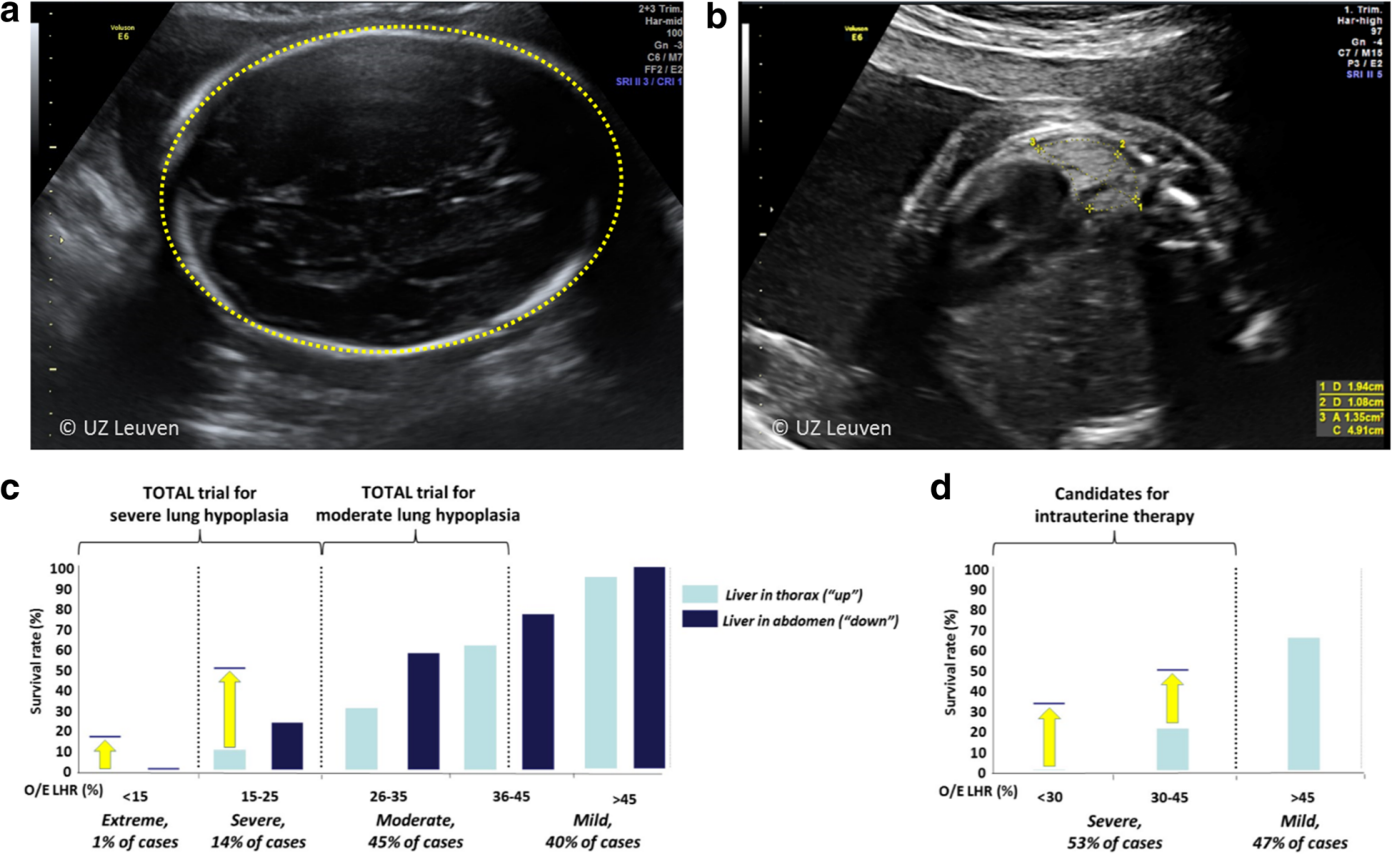
The o/e LHR is calculated by taking the ratio of the lung area divided by the head circumference, compared to a reference value for that gestational age. **a** Head circumference. **b** Lung area and diameter measured in the plane of the four-chamber view, the lung is posterior to the heart. **c** Survival rates of fetuses with left-sided CDH expectantly managed during pregnancy, as a function of different o/e LHR and liver position. **d** Same, for fetuses with right-sided CDH. Yellow arrows indicate improved survival after FETO as reported by Jani et al. 2006 (left CDH) and DeKoninck et al. 2014 (right CDH) [11, 26]. Adapted, with permission, from Russo et al. 2017 [27]

In isolated cases, personalized prediction of outcome is based on measurements of lung size, liver position, and the side of the defect [11]. Although other characteristics are being investigated among which stomach position, pulmonary circulatory parameters, and cardiac ventricular size as additional prognostic indicators, the lung-to-head-ratio (LHR) remains the best studied parameter for prediction. The lung contralateral to the lesion is measured in the standard plane for a four-chamber view of the heart, and the head circumference is measured in a standard biparietal view (Fig. 2a, b) [12]. The most accurate way of measuring the lung is by tracing its outline [13]. The LHR measured in the index case (observed) is expressed as a percentage (o/e LHR; %) of what one would expect in a normal fetus at a similar gestational age (expected). MRI is now almost ubiquitously used in tertiary centers for evaluation of congenital anomalies. In CDH cases, MRI allows for volumetric measurement of both lungs, the ability to quantitate the degree of liver herniation, and a detailed assessment of stomach position. So far, fetal MRI lung volumetry in CDH does not provide improved outcome prediction over ultrasound; hence, we do not use it for decisions regarding FETO. The predictive value of imaging methods in terms of pulmonary hypertension or extra-corporeal membrane oxygenation needs is currently under review [14].

Fetuses from singleton pregnancies with a predicted poor postnatal outcome are the target group for fetal intervention. The o/e LHR, presence of the liver in the thorax, and the side of the defect are used so stratify these fetuses [15] (Fig. 2c, d). Babies born beyond 30 weeks with a left-sided CDH, a herniated liver, and an o/e LHR < 25% who are managed with standard post-natal therapy have a survival rate that is < 20% [11]. These parameters were therefore used to define that group of fetuses with severe pulmonary hypoplasia, and they formed the initial study population for prenatal therapy. Despite encouraging early experience with FETO [16], fetal surgery for isolated CDH is still considered experimental. Patients with a fetus who has an isolated LCDH and *severe* lung hypoplasia are currently being offered participation in a global randomized clinical trial (https://www.totaltrial.eu/) comparing outcomes of FETO to expectant management during pregnancy, followed by standardized postnatal therapy [17]. In a second experimental arm to this trial, patients with a CDH fetus with *moderate* pulmonary hypoplasia (which has a 50% or greater survival rate) are randomized to FETO or expectant management, in an effort to reduce oxygen dependency at 6 months of age. For RCDH, fetal therapy is offered in case of severe hypoplasia (o/e LHR < 45%) because they have a predicted survival rate of 17%. For patients who have a more complex presentation with additional findings, a more individualized approach can be taken, yet in the absence of proof of benefit, this is debatable[18, 19].

### FETO or PLUG procedure

**Fig. 3:**
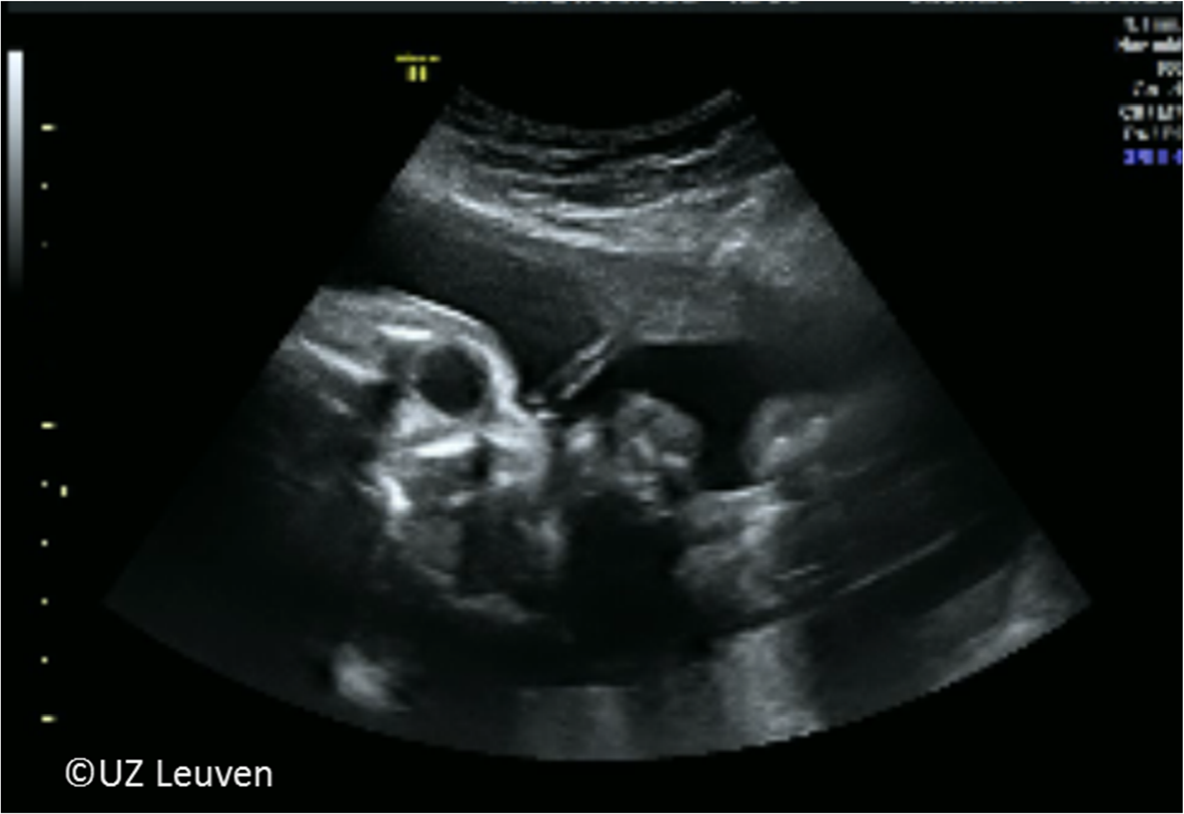
Transabdominal trocar entry in the direction of the tip of the nose. *© UZ Leuven, UZ Leuven, Belgium*


FETO was initially performed at 24–28 weeks only in cases of severe CDH and under epidural anesthesia. Today, the procedure is typically performed under local anesthesia at 27–29 weeks with conscious sedation optional, yet loco-regional can be done when clinically required. In moderate cases, FETO is done at 30–32 weeks. We use prophylactic tocolysis (atosiban or alternatively indomethacin or nifedipine) and antibiotics (cefazolin 2 g i.v. 8 hourly) until 24 h after the procedure. The patient is positioned in a dorsal supine position (with lateral tilt to prevent caval compression) such that there is direct access to the fetal mouth. External fetal manipulation may be required. Once in the appropriate position, we administer pancuronium (0.2 mg/kg) or equivalent, atropine (20 μg/kg), and fentanyl (15 μg/kg) intra-muscularly through a 22 G needle to the fetus to provide analgesia, immobilization, and prophylaxis against bradycardia. After sterile draping, the insertion trajectory is infiltrated with local anesthesia (lidocaine 1% 10–20 mL). A skin-incision is made and a disposable flexible 10 Fr cannula (3.3 mm; RCF 10.0 Check-Flo Performer, Cook, Bloomington, IN) loaded with a pyramidal trocar (11650TG, Karl Storz, Tutlingen, Germany) is inserted into the amniotic cavity under ultrasound guidance, in an area devoid of placenta and as perpendicular as possible to the nose tip (Additional file 1: Video S1; Fig. 3). Some introduce the cannula using the Seldinger technique [20].

**Additional file 1: Video S1.** The trocar is inserted into the amniotic cavity under ultrasound guidance, in an area devoid of placenta and as perpendicular as possible to the nose tip. Copyright: UZ Leuven, Leuven, Belgium. (MP4 4355 kb)


**Fig. 4:**
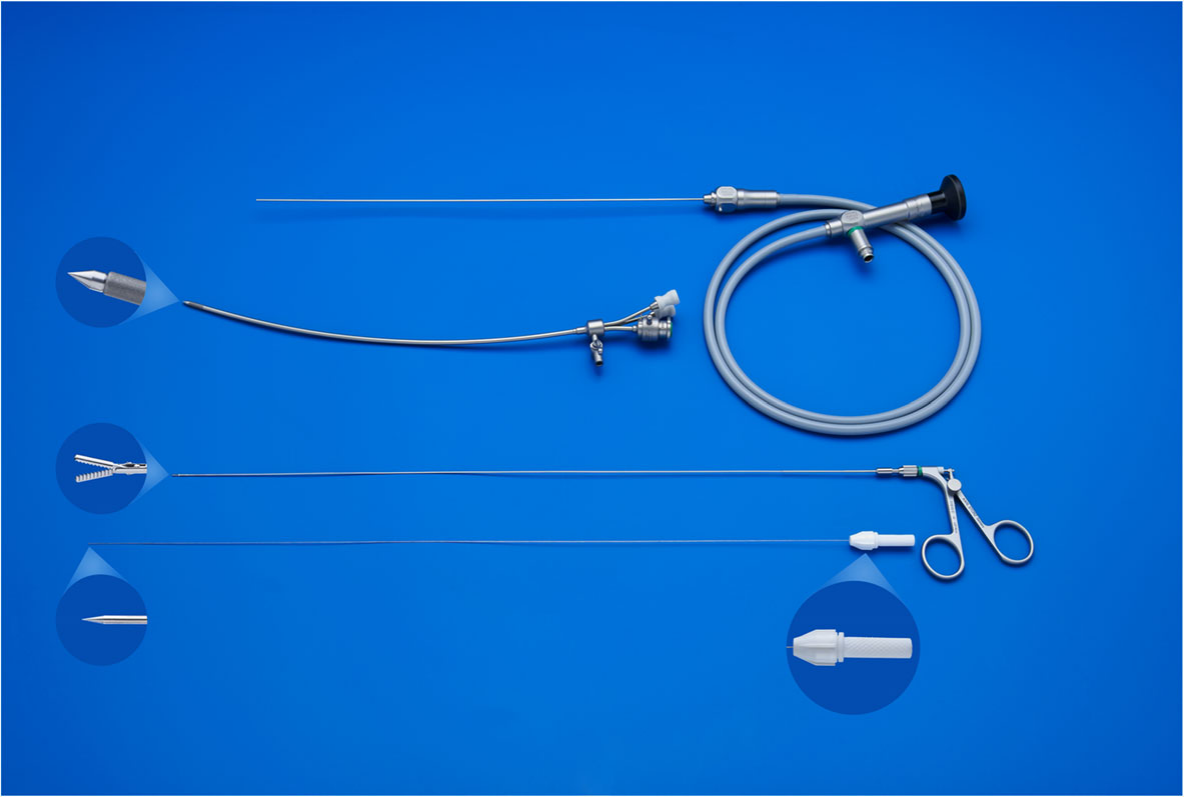
Fetoscope, fetoscopic forceps, and stylet, courtesy of *KARL STORZ Endoskope, Tuttlingen, Germany*

The fetoscope is a 1.3-mm fiber optic endoscope (11540AA, Karl Storz) housed within a curved 3.3-mm sheath (11540KE; Karl Storz) with a delivery catheter (Baltacci-BDPE-100 0.9 mm; Balt, Montmorency, France) that is loaded with a detachable inflatable latex balloon with integrated one-way valve (Goldbal2). These are used “off-label” as they were originally designed for endovascular occlusion. The inflated balloon accommodates for an increasing tracheal diameter during pregnancy. A stylet (11506P; Karl Storz) and grasping forceps (11510C, Karl Storz) are available to puncture and remove the balloon should it be incorrectly positioned (Fig. 4). We flush warmed crystalloid (Hartmann’s solution or Ringer’s Lactate) through the fetoscope sheath in order to distend the larynx, clear the debris, and improve vision.

**Fig. 5:**
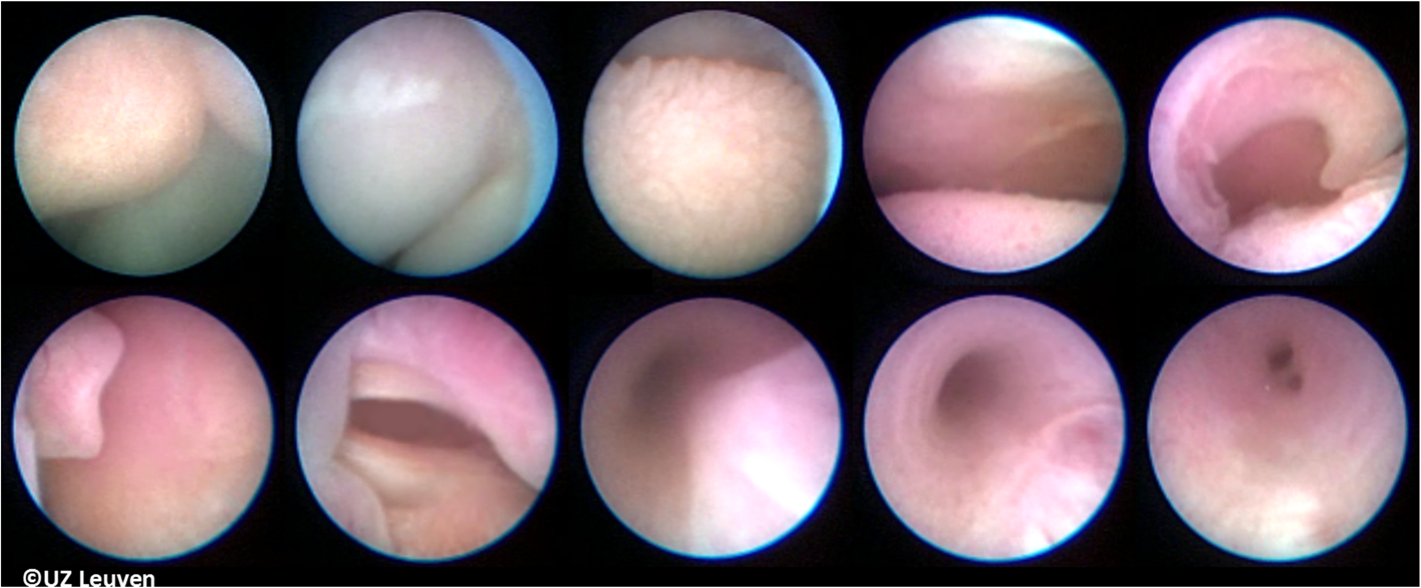
Landmarks used for guidance from the tip of the nose to trachea. Up from left to right: tip of nose, upper lip, tongue, raphe palate, and uvula. Down from left to right: epiglottis, vocal cords, trachea with inwards bulging pars membranacea, trachea more expanded and also better visualization of the tracheal rings, and carina. *© UZ Leuven, UZ Leuven, Belgium*

**Fig. 6:**
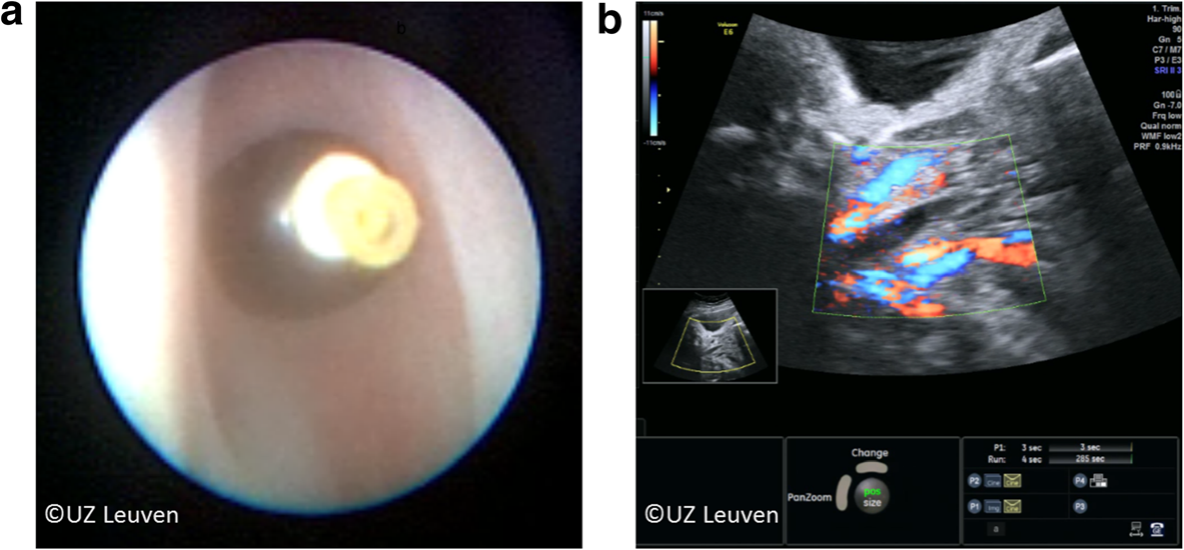
**a** After detachment the balloon can be seen through the vocal cords which are in this case just above it. **b** On ultrasound the balloon is visible as a hypoechogenic area. Power Doppler can help to distinguish the balloon from the adjacent blood vessels. *© UZ Leuven, UZ Leuven, Belgium*


Landmarks for balloon insertion are progressively the tip of the nose, philtrum, tongue, the raphe of the palate, uvula, epiglottis, and ultimately the vocal cords (Additional file 2: Video S2; Fig. 5). The fetoscope is advanced into the trachea until the carina is visualized, or if that is not possible, at least to a point where the tracheal rings and pars membranacea can be positively identified. The balloon is then advanced out of the fetoscope, positioned between the cords and the carina, and inflated with 0.6 mL of isotonic saline. Once inflated, the balloon is detached (Additional file 3: Video S3; Fig. 6a). Finally, excessive amniotic or irrigation fluid is drained until a normal volume is achieved. In our initial experience, the median duration of FETO was 10 min (range 3–93 min). Operating time depends mainly on operator experience and on the position of the fetus and is directly related to the risk of chorionic membrane separation and amniorrhexis.

**Additional file 2: Video S2.** The subsequent landmarks are seen as the fetoscope is advanced to the fetal trachea. Chronologically: lower lip, tongue, uvula, soft palate, epiglottis, vocal cords, and tracheal pars membranacea. Copyright: UZ Leuven, Leuven, Belgium. (AVI 4860 kb)



**Additional file 3: Video S3.** The balloon is inflated with 0.6 mL of isotonic saline solution and fills the trachea just distal to the cords, following which it is detached. Copyright: UZ Leuven, Leuven, Belgium. (AVI 6806 kb)


### Follow-up

Patients are followed with ultrasound every 1 to 2 weeks until the preset time for reversal of the occlusion. The fetus is evaluated for growth, movement, general wellbeing and routine antenatal care, and the cervical length is measured to estimate the risk for preterm birth. Amniotic fluid volume is measured to exclude polyhydramnios as this is common in CDH and can increase the risk of amniorrhexis and/or preterm labor. Amniodrainage is performed when the deepest vertical pool exceeds 12 cm. The membranes are inspected for amnion-chorion membrane separation. At each visit, the balloon is visualized since spontaneous deflation has been described [21]. The tracheal balloon appears on ultrasound as a hypoechoic fluid-filled structure without color Doppler flow and positioned just beneath the vocal cords between the common carotid arteries (Fig. 6b). Within a week following FETO, the fetal lung in responders becomes hyperechogenic. The parenchymal dimensional changes are quantified via the o/e LHR or MRI. Volume changes precede the vascular response. In case of amniorrhexis or preterm labor, the patients are admitted and management is individually planned for timely and safe balloon removal. Chorioamnionitis is the most common complication of membrane rupture and may mandate balloon removal and delivery. Antepartum hemorrhage has been described yet is in our experience uncommon [21].

### UNPLUG or balloon removal

**Fig. 7:**
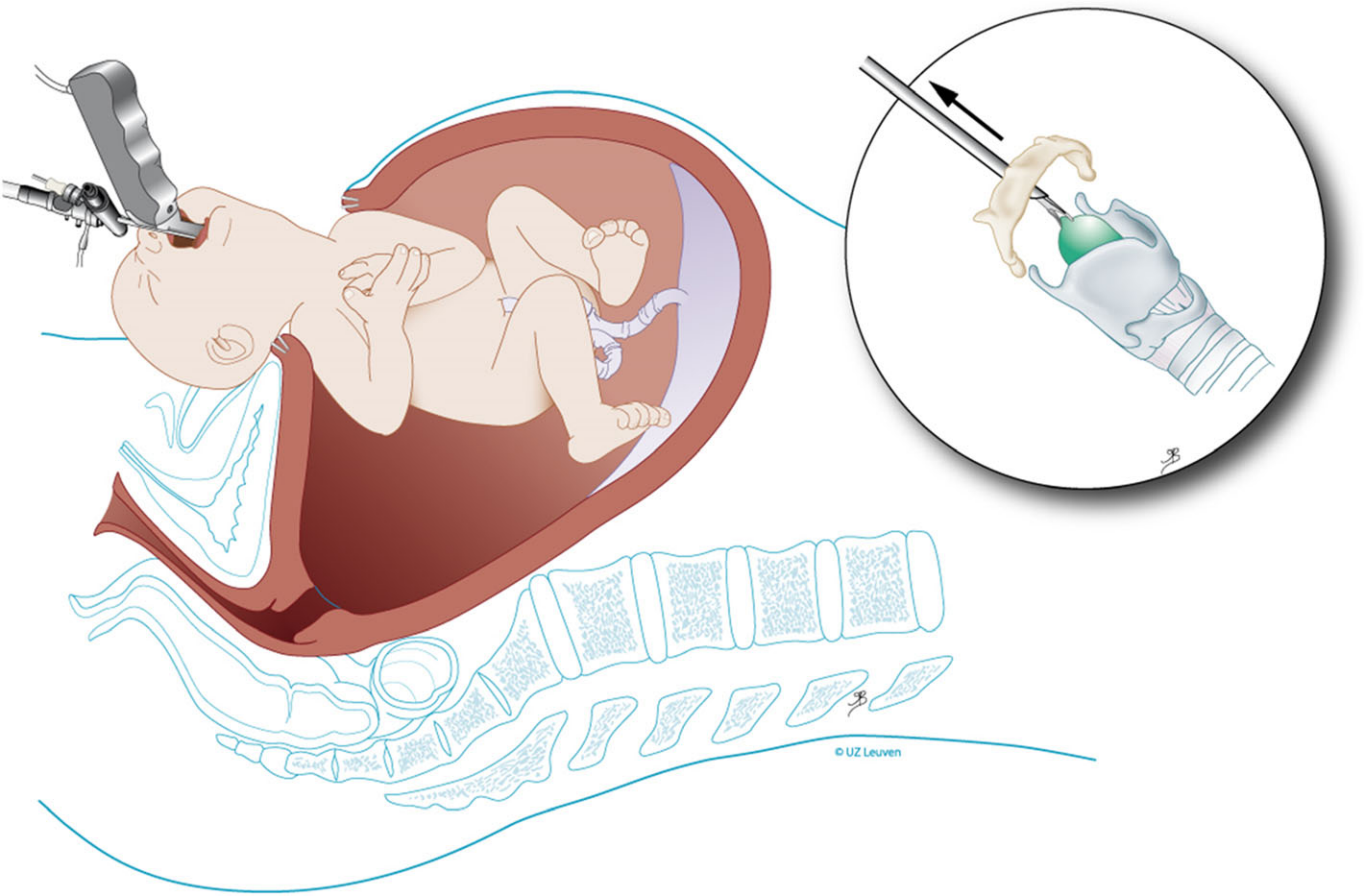
Schematic drawing of tracheoscopic removal on placental circulation under loco-regional anesthesia. *© UZ Leuven, UZ Leuven, Belgium, drawing Myrthe Boymans*


Our policy is to remove the balloon in utero even if delivery is imminent. It triggers lung maturation, increases survival chances, reduces morbidity [22, 23], and permits vaginal delivery. Removal of the balloon is scheduled at 34 weeks gestational age based on observations in sheep. In 28% of cases, balloon removal will be indicated earlier because of impending delivery [23]. Steroids are given to enhance lung maturation. The removal takes place in essentially the same preparation as described above for balloon placement, with the same fetal and maternal medications. The balloon can be punctured directly using a 20-22G needle under ultrasound guidance and in our experience is expulsed spontaneously. (Additional file 4: Video S4). In such cases, tracheal patency can be confirmed by demonstration of tracheal fluid movement with Doppler (Additional file 5: Video S5), change in tracheal diameter, or by MRI. The balloon can also be fetoscopically removed, which provides direct visualization of unobstructed airways (Additional file 6: Video S6). In the event that fetoscopic in utero removal is not possible, we resort to tracheoscopic removal (Table 1 for instruments) with the baby on placental circulation under loco-regional anesthesia (Fig. 7 and Additional file 7: Video S7). The fetal head and shoulders are delivered and direct laryngo-tracheoscopy is performed. In the worst (and not desirable) scenario, postnatal puncture from above the manubrium sterni is used, with or without ultrasound guidance, or by tracheoscopy. In a report on 302 cases, 67% of balloon removals were by fetoscopy, 21% by puncture, and 10% by tracheoscopy on placental circulation, and 1% ex utero [23]. In that study, the technique used appeared to be dictated mainly by operator preference. There was no difference in gestational age at delivery whether the balloon was punctured or removed by fetoscopy. The importance of immediate availability of trained and experienced operators who can remove a FETO balloon rapidly and safely cannot, and must not, be underestimated. To our knowledge, the only neonatal deaths directly due to balloon removal difficulties that have occurred happened when delivery took place in an unprepared and/or inexperienced environment [23].

**Table 1:** Overview of the fetoscopic instruments used for FETO and UNPLUG

Fetal tracheoscopy	Description	ID
1.3 mm endoscope	Miniature telescope, with remote eyepiece 0° straight forward, 30.6 cm working length	11540AA
3.3 mm sheath	Blunt curved sheath, with sand-blasted echogenic tip with stop cock for irrigation and two side openings	11540KE
1.0 mm forceps	Retrieval forceps, double action jaws, 35 cm long	11510C
0.4 mm stylet	Single use puncture stylet with adjustable torque, 50 cm long	11506P
0.9 mm needle	Puncture needle to protect the catheter or for aspiration, length 35 cm, can house the stylet	11540KD
3.3 mm trocar	10 Fr pyramidal tipped trocar for use with flexible cannula RCF-10.0 (Cook, Check Flo Performer)	11650TG
0.6 mL balloon	Goldbal 2 detachable latex balloon with radio-opaque inclusion, outer diameter 1.5 mm (inflated: 7.0 mm); length 5.0 mm (inflated 20.0 mm)	Goldbal 2 (Balt)
0.9 mm microcatheter	catheter loaded with mandrel, and Touhy Boost Y-connection, max outer diameter 0.9 mm, tapered to 0.4 mm, 100 cm in length	“Baltacci” e BDPE 100 (Balt)
Direct bronchoscopy 1.3 mm endoscope	Miniature telescope, with remote eyepiece 0° straight forward, 18.8 cm working length	10040AA
Straight bronchoscopic sheath	4.2 mm outer, 3.5 mm inner diameter 18.5 cm length (size 2.5), is conventional neonatal “Doesel-Huzly” bronchoscope, with blanking and suction plug	10339F 10924SP 10315RV
Telescope bridge	Houses telescope and has side opening for irrigation 1.5 mm outer diameter	10338LCI
1.0 mm forceps	19 cm semi-flexible forceps for balloon retrieval	10338H
0.4 mm stylet	Single use puncture stylet with adjustable torque, 50 cm long	11506P

Endoscopic instruments were developed by Karl Storz Endoskope, supported by the European Commission in the 6th framework program. The balloon system is an adapted version of a commercially available vascular occlusion device. Most instruments and devices are used off label

**Additional file 4: Video S4.** The balloon is punctured under ultrasound guidance using a 20–22 G needle. The needle is inserted as close as possible to the anterior shoulder, which enables to pass the umbilical cord which is lying on the neck. As the balloon is punctured, the trachea can be seen collapsing. Copyright: UZ Leuven, Leuven, Belgium. (MP4 5472 kb)



**Additional file 5: Video S5.** After puncturing, the balloon tracheal patency can be confirmed by demonstration free tracheal fluid movement with Doppler. Copyright: UZ Leuven, Leuven, Belgium. (MP4 3849 kb)



**Additional file 6: Video S6.** Fetoscopic unplugging provides direct documentation of free airways. As the lung fluid egresses, the pressure drops and the trachea will slightly collapse. Copyright: UZ Leuven, Leuven, Belgium. (AVI 2303 kb)



**Additional file 7: Video S7.** If not possible to puncture the balloon ultrasound guided or fetoscopic guided, we can resort to tracheoscopic removal on placental circulation under loco-regional anesthesia. Copyright: UZ Leuven, Leuven, Belgium. (MP4 6707 kb)


Reported adverse events and side effects with FETO are rare. Fetal tracheomegaly is a recognized entity that usually presents as a barking cough on effort and then resolves over time with minimal long-term implications. There are a few neonates who have had significant long-term tracheal issues, and these appear to have been related to traumatic balloon removal or early insertion < 26 weeks [24]. This is however an aspect that requires more detailed and long-term follow-up. The main maternal-fetal complication is that of chorionic membrane separation and PPROM with resultant preterm birth. Although the median gestational age at birth is 35 weeks, up to one in three patients deliver prior to 34 weeks, potentially offsetting the effect of the fetal intervention.

## Results

To date, the published data are predominantly based on observational trials and small case series [8]. There is one randomized controlled trial [25, 26] that showed benefit of FETO; however, in that study all deliveries were by EXIT procedure, there was a case mix of right and left CDH, and the methodology was substantially different to what is currently being practiced in Europe and the USA.

Compared to historical controls of similar severity, FETO increases survival rate from 24 to 49% in LCDH with o/eLHR < 25% and from 17 to 42% in RCDH with o/eLHR < 45% [21, 26] (Fig. 2c, d). FETO also seems to reduce early neonatal respiratory morbidity.

## Discussion

This potential benefit is now being investigated in two parallel randomized clinical trials (RCT) “Tracheal Occlusion To Accelerate Lung growth” (https://www.totaltrial.eu/), in fetuses with LCDH and either severe or moderate lung hypoplasia (NCT01240057 and NCT00763737). Current fetal treatment centers are in Leuven, Belgium; Paris, France; London, UK; Barcelona, Spain; Milan and Rome, Italy; Bonn, Germany; Toronto, Canada; Brisbane, Australia; and Houston, Texas, USA. These centers have a high volume fetoscopy program, completed a minimum learning curve of 15 FETO procedures, and committed to strict adherence to a prenatal and postnatal management protocol. The trial has recruited at the time of writing over 165 (moderate) and over 50 (severe) patients. Results are expected within the next 2 years at the current recruitment rate (moderate). We anticipate that more centers in the USA and in Japan will be joining the task force which may accelerate recruitment.

## Conclusions

FETO may alter the natural history of congenital diaphragmatic hernia, and early clinical results look promising. It is hoped that the ongoing TOTAL trial will result in proof of benefit. FETO is an invasive technique associated with a significantly increased risk for preterm birth which potentially tempers its benefits. The procedure requires specific skills and instrumentation and permanent services and is at present limited to a select group of centers. If proven effective, this procedure is likely to be implemented more widely and appropriate dissemination will require an extensive training program and careful oversight in order to ensure safe implementation.

## Abbreviations

CDH: Congenital diaphragmatic hernia
EXIT: Ex utero intrapartum treatment
FETO: Fetal endoluminal tracheal occlusion
LCDH: Left-sided congenital diaphragmatic hernia
LHR: Lung-to-head-ratio
MRI: Magnetic resonance imaging
o/e LHR: Observed to expected lung-to-head-ratio
PLUG: Plug the Lung Until it Grows
PPROM: Preterm and Prelabor Rupture Of Membranes
RCDH: Right-sided congenital diaphragmatic hernia
RCT: Randomized clinical trial
TOTAL: Tracheal Occlusion To Accelerate Lung growth
